# Time‐Resolved Encryption via a Kinetics‐Tunable Supramolecular Photochromic System

**DOI:** 10.1002/advs.202104790

**Published:** 2022-01-06

**Authors:** Dong Li, Zefen Feng, Yujie Han, Chen Chen, Qi‐Wei Zhang, Yang Tian

**Affiliations:** ^1^ Shanghai Key Laboratory of Green Chemistry and Chemical Processes Department of Chemistry School of Chemistry and Molecular Engineering East China Normal University Shanghai 200241 P. R. China

**Keywords:** cucurbit[8]uril, dynamic encryption, photochromism, supramolecular systems

## Abstract

With the advancement of forgery and decryption methods, conventional static encryption technology is becoming more and more powerless, which strongly demands the development of multistate anticounterfeiting materials as well as advanced multidimensional encryption strategies and technologies. Here a new strategy to realize time‐resolved encryption based on a self‐assembled supramolecular ternary complex is presented, which exhibits tunable dynamic photochromic features caused by the reversible photodimerization/cleavage reactions of the guest chromophores inside the cavity of cucurbit[8]uril (CB[8]). This supramolecular system shows excellent photochromic properties, including extremely rapid response time, high conversion rate, and product‐stereoselectivity, etc. More importantly, the kinetics of the photoreaction can be modulated by simply varying the host–guest ratios in aqueous or quasi‐solid phase, providing the material with finely tunable time‐dependent features, which cannot only be employed in data processing with more extended information, but also construct confidential materials by time‐resolved multidimensional encryption and dynamic anticounterfeiting. The strategic design of kinetics‐tunable supramolecular photochromic materials may provide valuable guidance for the development of more advanced materials for information security.

## Graphical abstract

A smart kinetics‐tunable supramolecular photochromic system based on a macrocycle‐catalyzed and ‐modulated reversible photodimerization reaction is reported. This concise yet powerful supramolecular platform provides a time‐involved dimensionality‐increase approach to comprehensively combine the static and dynamic characteristics of matters, which will greatly improve the capacity of information processing and render the application of multidimensional dynamic encryption with much higher security.

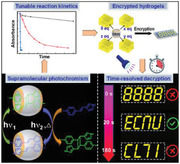

## 1 Introduction

Forgery and cracking of secure and confidential documents or information, including passports, certificates, bank checks, passwords, etc., have occurred frequently in various occasions, which brings huge security and property risks to governments, companies, and individuals. Therefore, the development of advanced anticounterfeiting materials and encryption technologies to improve information security has become a global issue and a great challenge for informationists, chemists and materials scientists.^[^ ^1^, ^2^, ^3^, ^4^, ^5^ ^]^ To date, a series of anticounterfeiting materials with magnetic, electronic or optic properties have been developed, among which optical materials offer superiority in accurate design, high throughput, and easy handling.^[^ ^6^, ^7^ ^]^ However, most of the traditional materials are based on their static characteristics or signals, which greatly limit their advanced applications in information encryption. With this in mind, materials with multiple states that can be switched under external stimuli on demand would be promising candidates.^[^ ^8^, ^9^, ^10^, ^11^, ^12^ ^]^ Photochromic compounds as an important class of stimuli‐responsive materials, have attracted an explosion of interest in recent years.^[^ ^13^, ^14^, ^15^, ^16^ ^]^ These compounds are able to display distinct colors in response to external photochemical or additional thermal stimuli via reversible isomerization, cyclization, or cycloaddition reactions, during which, molecular structures with different geometry, conjugations and optical features are achieved.^[^ ^17^, ^18^ ^]^ The most studied photochromic compounds are diazobenzenes, diarylethenes, spiropyrans, etc., whose switchable colors, polarities, and emission properties cause their vast applications in the fields of molecular switches, logic gates, as well as anticounterfeiting and encryption.^[^ ^19^ ^]^ However, in most cases, only the two states of photochromic materials are employed in those fields, so the capacity of information storage, data processing, and encryption is still limited. In order to address these confinements, a powerful strategy is to develop kinetics‐tunable photochromic materials that comprehensively utilize the static and dynamic characteristics of matters, which will greatly improve the capacity of information processing and render the application of multidimensional encryption with much higher security.^[^ ^20^ ^]^

Photodimerizations of alkene‐ or acene‐contained chromophores as a special type of photochromic processes, which differs from those intramolecular photochromic reactions such as photoisomerization and ring‐opening/closing, are based on the intermolecular cycloadditions.^[^ ^21^, ^22^ ^]^ Due to their intermolecular nature, the reaction kinetics and thermodynamics are often susceptible to the influence of the surrounding environment and spatial arrangement of the chromophores.^[^ ^23^, ^24^ ^]^ From this aspect, noncovalent interactions, such as hydrogen bonding, host–guest effects, electrostatic attraction, *etc*., may play important roles owing to their ability to sequester the substrates in a preorganized configuration or packing, thereby lowering the activation energy for the photodimerization and subsequently expelling the dimer products due to the less favorable new geometries or interactions.^[^ ^25^, ^26^ ^]^ For example, the regio‐ and stereoselective photodimerization of anthracene, coumarin or merocyanine derivatives have been achieved within supramolecular hosts such as cyclodextrins, cucurbiturils, metal–organic frameworks, and other macrocycles or cages, benefiting from their rigid and confined cavities.^[^ ^27^, ^28^, ^29^, ^30^, ^31^, ^32^, ^33^ ^]^

**Scheme 1:**
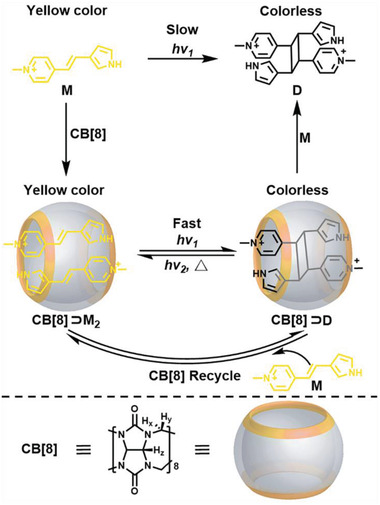
Structures of the compounds and schematic diagram of the CB[8]‐manipulated photochromic processes based on the reversible photodimerization/cleavage reactions. The counter anions of chloride have been omitted for clarity.

Herein, we reported an example in which an pyrrylethenyl pyridinium derivative (named **M**, see **Scheme** **1**) was able to form a 1:2 host–guest self‐assembled structure with cucurbit[8]uril (CB[8]) in aqueous solution, stabilized by the cooperativity of a series of supramolecular interactions, including the cation‐*π* interaction between substrates, the charge–dipole interactions and hydrophobic effects between host and guest, etc.^[^ ^34^, ^35^ ^]^ In this supramolecular ternary complex, an effective and reversible photodimerization can occur under UV irradiation, with very high reaction conversion, product‐stereoselectivity, and reversibility. Meanwhile, the color of the sample changed from yellow to colorless, showing a kind of negative photochromism.^[^ ^36^, ^37^ ^]^ More importantly, the reaction rate of this photodimerization reaction can be modulated by controlling the amount of CB[8] macrocycles in the host–guest systems, in this way, a time‐dimension could be introduced into the process of signal interpretation based on this unique photochromic material. In principle, this time‐involved dynamic strategy is more competitive over those static approaches, because it can extend the data density from sequence level to a matrix level, thereby vastly increasing the capacity of information storage and processing. What's more, a proof‐of‐concept time‐resolved encryption technology was practically developed, providing a guiding design for more security materials in the future, in which encrypted codes can only be read out at a correct time node.

## 2 Results and Discussion

### 2.1 Host–Guest Self‐Assembly

**Figure 1:**
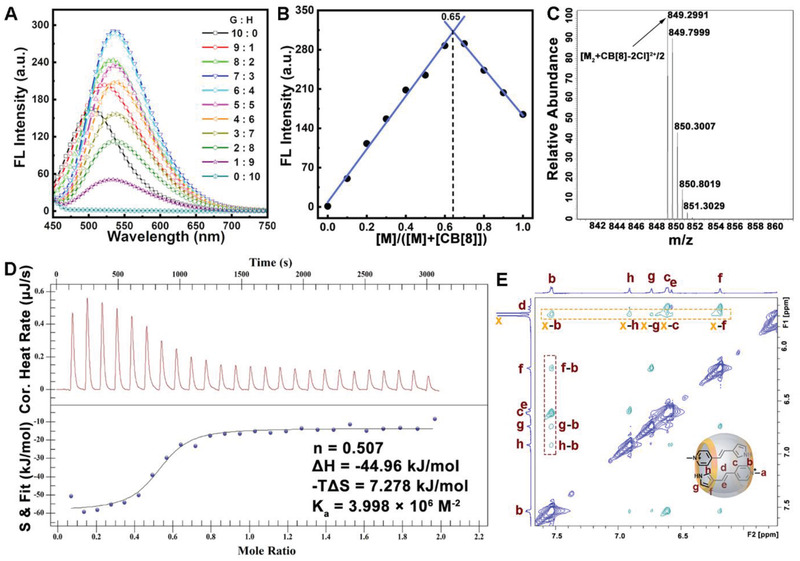
A) Fluorescence spectrum of the guest molecule **M** and host CB[8] mixtures in aqueous solution with difference host:guest ratios (the total concentration was fixed as 10 × 10^−6^ m, at room temperature, excited at 390 nm). B) Job's plot for the complexation between **M** and CB[8] based on the fluorescence intensity changes at 538 nm in spectrum (A). C) Mass spectrometry of the complex CB[8]⊃**M**_2_. D) ITC spectrum for the binding between **M** and CB[8] in water, [**M**] (cell) = 10 × 10^−6^ m, CB[8] (syringe) = 100 × 10^−6^ m. E) Partial 2D ROESY NMR spectrum of the complex CB[8]⊃**M**_2_, in which [**M**] = 2 [CB[8]] = 2 × 10^−3^ m in D_2_O at 298 K.

As shown in Scheme 1, the water‐soluble chromophore **M** was designed and synthesized conveniently by the Knoevenagel condensation between 1,4‐dimethylpyridinium and 3‐formylpyrrole at room temperature, which was selected as a potential photochromic molecule in aqueous system due to its moderate water‐solubility and the possible photodimerization capacity. On the other hand, the macrocycle CB[8], which bears a rigid and hydrophobic cavity as well as two polar portals with strong electronegativity, was chosen as the host molecule owing to its potential to accommodate two hydrophobic or positively charged aromatic molecules.^[^ ^38^, ^39^ ^]^ In order to obtain the binding features between the guest molecule **M** and the host molecule CB[8], fluorescence emission experiments were initially carried out with different host/guest ratios in aqueous solution (**Figure** **1**A). The corresponding Job's plot presented in Figure 1B clearly suggested a 1:2 host–guest binding stoichiometry. Besides, the complex formed between CB[8] and **M** exhibited a red‐shifted emission compared to the monomer **M**, indicating the possible cation‐*π* interaction between the pyridinium and aromatic moieties of the adjacent molecules inside the cavity of CB[8], which may further induce the intermolecular charge transfer emission with red‐shifted wavelength. The formation of a ternary supramolecular complex (named CB[8]⊃**M**_2_, see Scheme 1) was also evidenced by ESI‐mass spectrometry (Figure 1C), in which a strong peak belong to a mass‐to‐charge ratio (*m*/*z*) of 849.2991 for [**M**_2_+CB[8]‐2Cl]^2+^/2 was observed. In addition, the isothermal titration calorimetry (ITC) experiment was further performed in aqueous solution to study the thermodynamic characters of the supramolecular host–guest complexation between **M** and CB[8]. As shown in Figure 1D, the fitting curve of the titration data again confirmed the 1:2 binding mode (the *n*‐value is measured as 0.5). The overall binding constant (*K*_a_) was evaluated to be 3.998 × 10^6^ m ^–2^, indicating the strong affinity between the host and guest molecules in water, which is mainly driven by the favorable enthalpic contributions (Δ*H* = −44.96 kJ mol^−1^).

To get detailed structural characterization of the supramolecular complex CB[8]⊃**M**_2_, the NMR resonances of the guest molecule was firstly assigned with the aid of 2D ^1^H‐^1^H COSY and ROESY NMR techniques (Figures S1 and S2, in the Supporting Information). Based on these unequivocal assignments of the protons of **M**, we were able to study the self‐assembly behavior through NMR experiments. As shown in Figure S3b (Supporting Information), the ^1^H NMR spectrum for the 1:2 mixture of macrocycle CB[8] and monomer **M** in D_2_O shows a completely different pattern from the respective components (Figure S3a,c, Supporting Information). All the aromatic protons from the pyridine and pyrrole moieties, as well as the two sets of protons from the alkene group of **M** shifted upfield dramatically when mixed with 0.5 equivalence of CB[8], indicating the strong shielding effect induced by the encapsulation of **M** into the cavity of CB[8]. Furthermore, 2D ROESY NMR was additionally measured to obtain more detailed geometrical information of the host–guest complex CB[8]⊃**M**_2_. As shown in Figure 1E, a series of rotating‐frame nuclear Overhauser effects (NOE) signals were found between the aromatic protons H_b_, H_h_, H_g_, H_c_, H_f_ of the guest molecule **M** and the inward methylene proton H*_x_* of CB[8] (see the dotted frame in orange color), confirming the encapsulation of **M** within the cavity of CB[8]. What's more, another set of cross peaks could be found by further investigation, such as the correlated signals H_f_‐H_b_, H_g_‐H_b_, and H_h_‐H_b_ marked by the red dotted frame in Figure 1E. The close proximity between these protons at both ends of the linear and rigid compound **M** clearly evidenced the head‐to‐tail (H‐T) stacking mode between the two guest molecules inside the cavity of CB[8], as illustrated in Scheme 1.

### 2.2 Photochemical Behaviors of the Supramolecular Ternary Complex

**Figure 2:**
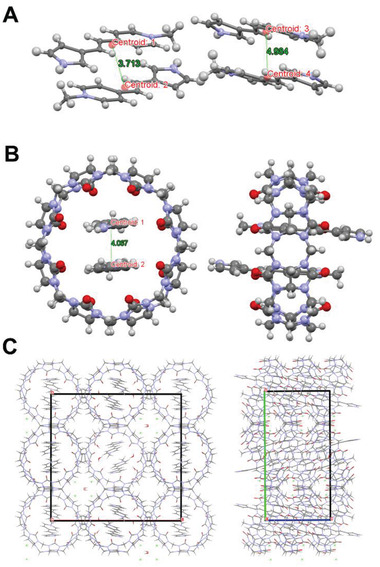
A) X‐Ray single crystal structure and two intermolecular stacking modes of the guest molecule **M**. B) X‐Ray single crystal structure and C) the intermolecular stacking of the 1:2 host–guest complex CB[8]⊃**M**_2_. The counteranions were omitted for clarity.

Basically, photodimerization is a reaction where two adjacent chromophores are aligned in a well‐organized stacking for the topochemical cycloaddition with a close center‐to‐center distance. As a result, the geometry of the molecular structure, the intermolecular interactions, and the stacking orientation may play crucial roles in the photodimerization process. To get these information directly for **M**, we successfully grew two single crystals for the monomer **M** (CCDC NO. 2103177, Table S1, Supporting Information) and the complex CB[8]⊃**M**_2_ (CCDC NO. 2116478, Table S2, Supporting Information) by slow volatilization of their aqueous solutions, which were subsequently analyzed crystallographically. As shown in **Figure** **2**A, the as synthesized chromophore **M** is in a *trans*‐configuration with a relatively coplanar structure. The width of the structure was measured to be ≈4.3 Å, which meets the prerequisite for the possible encapsulation within CB[8] (the diameter of the portal is 6.9 Å).^[^ ^38^ ^]^ Besides, two kinds of intermolecular antiparallel (head‐to‐tail) stacking modes were found in the single crystal structure of **M** with the centroid distances of 3.713 and 4.984 Å, respectively (for a larger‐range stacking view, see Figure S4 in the Supporting Information). Such stacking type in the solid state may be induced by the cation‐*π* interactions between the pyridinium and aromatic moieties of the adjacent molecules, which could provide a favorable preorganization for the formation of a ternary complex with CB[8], as illustrated in Scheme 1.^[^ ^40^ ^]^ On the other hand, a more regular molecular stacking could be observed for the supramolecular complex CB[8]⊃**M**_2_ in the single crystal structure (Figure 2B). Two guest molecules are encapsulated inside the cavity of CB[8] in a head‐to‐tail antiparallel manner with a uniform centroid distance of 4.067 Å, and this supramolecular geometry is consistent with the optical, mass and NMR analyses mentioned above. This well‐organization in the cavity not only caused the intermolecular charge transfer process between the two guest molecules, which was verified by the red‐shifted absorption of **M** upon addition of CB[8] (Figure S5, Supporting Information). In addition, the antiparallel arrangement and short centroid distance in the cavity of CB[8] also provides a structural basis for the high efficiency and high stereoselectivity of the photodimerization of **M**, which will be discussed in the following contents.

**Figure 3:**
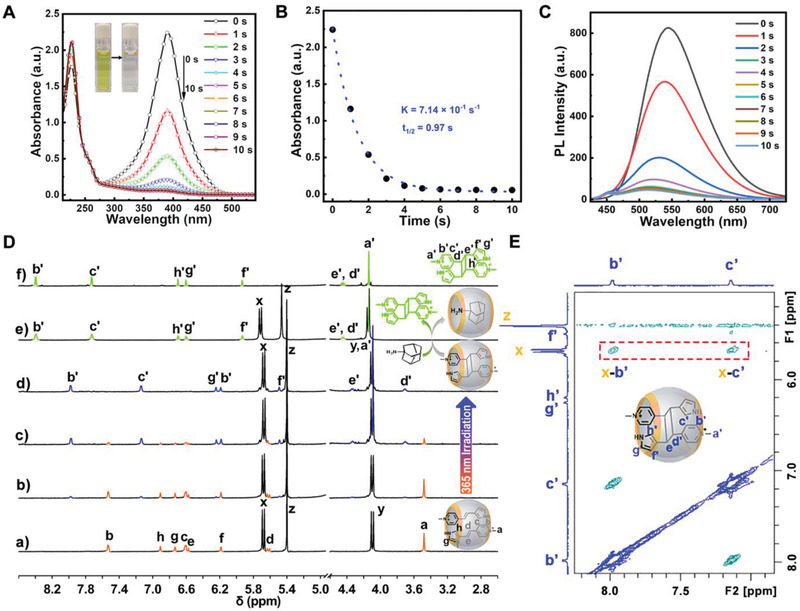
A) Absorption spectra changes of the aqueous CB[8]⊃**M**_2_ solution ([**M**] = 100 × 10^−6^ m) upon irradiation with 365 nm UV light for different time (0–10 s). Inset is the daylight pictures of the sample solution before (left) and after (right) 10 s of UV irradiation. B) the corresponding change of absorbance at 390 nm (black circles) at different irradiation time, and fitted with the first‐order kinetic model (blue dash line). C) Fluorescence spectra changes of the same sample and condition in panel A (excited at 390 nm). D) ^1^H NMR spectra of a) the complex CB[8]⊃**M**_2_ ([**M**] = 2 × 10^−3^ m) in D_2_O, and irradiated with 365 nm UV light for b) 5 s, c) 10 s, and d) 20 s (formed the pure photodimerized complex CB[8]⊃**D**), e) the complex CB[8]⊃**D** upon addition of one equivalence of **Ad**, by which the free dimer **D** and the new complex CB[8]⊃**Ad** were formed (the peaks for **Ad** are not shown, which locate at around 1.5 ppm), and f) the free dimer **D** obtained by direct irradiation of the **M** solution in D_2_O with 365 nm UV light for 50 min. E) Partial 2D ROESY NMR spectrum of the complex CB[8]⊃**D** in D_2_O at 298 K.

After the structural characterizations of both the monomer **M** and the host–guest complex CB[8]⊃**M**_2_, we then explored the effect of UV irradiation on the photoreactions of these two systems comparatively in aqueous solution. The response of CB[8]⊃**M**_2_ to 365 nm UV light irradiation was firstly investigated by absorption spectrometry. As shown in **Figure** **3**A, the absorbance intensity at 390 nm decreased dramatically and rapidly upon irradiation, which could reach the photostationary state within 10 s with a high conversion rate of ≈98%. Moreover, the intensity attenuation of the absorption peak at 390 nm could be fitted by the first‐order kinetics (Figure 3B, *k* = 7.14 × 10 ^–1^ s^–1^, *t*_1/2_ = 0.97 s). Such fast reaction rate and the fairly fitted first‐order kinetic character were ascribed to the encapsulation of **M** into the cavity of CB[8], because the reaction rate for the pure **M** solution was found to be much slower when irradiated at the same condition but without CB[8] (Figure S6, Supporting Information). In addition, as the irradiation progressed, the color of the solution changed from yellow to colorless, and the yellow emission also disappeared simultaneously (see Figure 3A, inset, Figure 3C, and the Movie S1 in the Supporting Information), exhibiting a kind of negative photochromism.

Taking into account of the possible different types of photochemical reactions, such as *cis/trans*‐isomerization and photodimerization of diarylethene derivatives, here in order to further confirm the mechanism and products of the photoreaction, we performed the photoreaction of **M** solution in D_2_O, and tracked the chemical shifts of the protons during UV irradiation in situ by NMR technology. As shown in Figure S7 (Supporting Information), the chromophore **M** existed purely in *trans*‐configuration before UV irradiation. In the initial 25 min of irradiation, a small portion of isomerized products may exist according to the new small peaks around 7.93 ppm, however, when extended the irradiation to 50 minutes, a relatively pure photodimerization product **D** was obtained with a conversion rate of ≈88%. As a control, with the presence of CB[8] (0.5 equivalence), the photodimerization of **M** was significantly accelerated at the same conditions, which could reach the photostationary state within 20 s with almost quantitative conversion to a syn‐HT configuration (Figure 3D‐a–d).

More interestingly, in the case of irradiation the mixture solution (CB[8]:**M** = 1:2), all the photodimerized product (**D**) was found to be still entrapped in the cavity of CB[8] (formed the binary complex CB[8]⊃**D**), which was confirmed by the higher field chemical shifts of all the protons of **D** in the host–guest mixture (Figure 3D‐d) than that in the free state (Figure 3D‐f). Besides, the NOE signals between the inward methylene proton H*_x_* of CB[8] and the aromatic protons H_b’_, H_c’_ of the dimer **D** in the ROESY NMR spectrum further confirmed this host–guest binding (Figure 3E). However, if another competitive guest molecule Amantadine (**Ad**) was added to the CB[8]⊃**D** solution, the dimer **D** could be drained out of the macrocycle and stay in a free state in solution due to the stronger binding affinity between **Ad** and CB[8].^[^ ^38^ ^]^ This guest‐replacement could be proved by the fact that all the chemical shifts of the protons of **D** in the mixture returned back to the lower field, which exhibited exactly the same chemical shifts as the free dimer (**D**) that was produced by directly irradiation of **M** solution (see the green peaks in Figure 3D‐e,f). By further investigation, we found that such guest‐exchange process of the complex CB[8]⊃**D** in water not only existed upon addition of **Ad**, but also could be driven by the excess monomer **M** itself. As shown in Figure S8 (Supporting Information), when a small amount of **M** was added to the pure CB[8]⊃**D** solution in D_2_O, two sets of NMR peaks could be observed (Figure S8b, Supporting Information), which could be doubtlessly assigned to the free dimer **D** and the self‐assembled ternary complex CB[8]⊃**M**_2_ respectively, by comparison with the corresponding pure samples (Figure S8a,c, Supporting Information). This result clearly confirmed the role of CB[8] as a supramolecular catalyst, which was able to recycle spontaneously and continuously when excess of **M** exists in the system.

### 2.3 Applications of the Supramolecular Photochromic System

**Figure 4:**
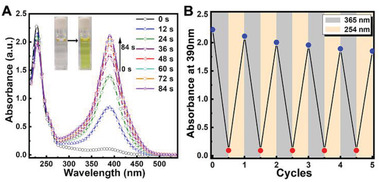
A) Absorption spectra changes of the aqueous CB[8]⊃**D** solution ([**D**] = 50 × 10^−6^ m) upon irradiation with 254 nm UV light at 60 °C for different time (0–84 s). Inset is the daylight pictures of the sample solution before (left) and after (right) 84 s of UV irradiation. B) Intensity changes of the absorption at 390 nm for the complex CB[8]⊃**M**_2_ in aqueous solution upon alternating UV lights irradiation (365 nm, 10 s; and 254 nm, 84 s at 60 °C).

Photochromic molecules, as a kind of smart material with potential applications, their properties such as the response time, conversion rate, and the switching reversibility are the main considerations. However, due to the nature of the intermolecular reaction of dimerization and the possible competitive side photoreactions, the normal photochromic systems based on photodimerization mechanism often suffer from the low conversion rate, low product purity, and long response time.^[^ ^23^ ^]^ What is worse, because the photocleavage reaction needs to break two *σ*‐bonds simultaneously, thus requiring high energy of irradiation, in which wavelength an absorption equilibrium may exist for the reactants and products. As a result, the reverse reaction may be even less efficient. Luckily, the supramolecular system developed here (CB[8]⊃**M**_2_) was found to have excellent performance in those aspects. As mentioned above, the irradiation of CB[8]⊃**M**_2_ aqueous solution ([**M**] = 100 × 10^−6^ m) with 365 nm UV light could lead to the pure dimer product CB[8]⊃**D** almost quantitatively in 10 s. On the other hand, the reverse photocleavage process could be achieved by exposure to 254 nm UV light irradiation at 60 °C, with a conversion rate of ≈95% in 84 s (**Figure** **4**A). The kinetics of the cleavage reaction are fitted by a first‐order reaction equation with a reaction rate coefficient of *k* = 4.6 × 10 ^–2^ s^–1^, and *t*_1/2_ = 15.05 s (Figure S9, Supporting Information). The reversible photodimerization/cleavage processes could be repeated effectively for a series of cycles between the two photostationary states (Figure 4B). It is worth mentioning that if the switching is operated within the irradiation time for a conversion of around 75%, excellent reversible cycles can be obtained (Figure S10, Supporting Information). On the other hand, no reverse‐reaction or only very low conversion could be observed, when the sample is heated without light or irradiated at room temperature, respectively (Figure S11, Supporting Information).

**Figure 5:**
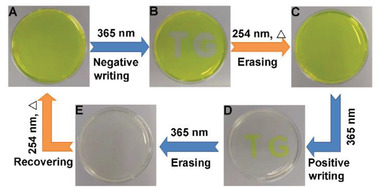
A) Photograph of CB[8]⊃**M**_2_‐doped agarose hydrogel. B) Two colorless letters “TG” were written on the hydrogels by irradiation with 365 nm light using a mask. C) The colorless letters “TG” were erased by 254 nm UV light irradiation. D) The new yellow‐colored letters “TG” with a colorless background were obtained by masking the letters while irradiating the rest of the area. E) The yellow‐colored letters “TG” were further erased by 365 nm UV light irradiation. Finally, the yellow‐colored hydrogel could be recovered.

Inspired by the above results, we then initially explored the potential applicability of CB[8]⊃**M**_2_ as a photoresponsive rewritable material in a quasi‐solid state. A yellow hydrogel could be conveniently obtained by simply dispersion the agarose to the CB[8]⊃**M**_2_ aqueous solution (**Figure** **5**A). It is worth noting that the supramolecular photochromic properties induced by the reversible dimerization/cleavage reactions were well maintained in this hydrogel state. As shown in Figure 5B, two colorless letters “TG” were written on the CB[8]⊃**M**_2_‐doped yellow hydrogel immediately by 365 nm UV light irradiation for 20 s (called as the “negative writing” process), which could be erased after 254 nm light irradiation (Figure 5C). Moreover, the new yellow‐colored letters “TG” with a colorless background were obtained by masking the letters while irradiating the rest of the area (Figure 5D), which could be further erased by continuing irradiation after removing the mask (Figure 5E). Finally, the yellow‐colored hydrogel could be recovered. Such reversible write‐erase processes have verified its basic practical application as a supramolecular photochromic material in hydrogel state.

**Figure 6:**
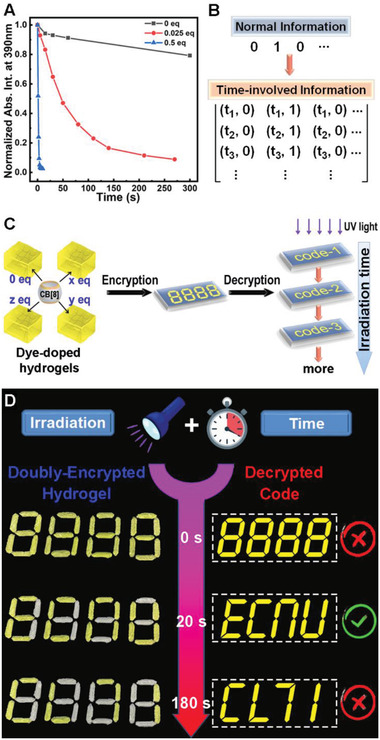
A) Diagram of the absorption intensities at 390 nm versus irradiation time of 365 nm UV light of the aqueous **M** solution with varied equivalences of CB[8], [**M**] = 100 × 10^−6^ m. B) Schematic illustration of a normal sequential digital information and the time‐contained matrix‐like digital information. C) Schematic illustration of the encryption–decryption principle using different hydrogels based on CB[8]⊃**M**_2_. D) The practical application of the CB[8]⊃**M**_2_‐based hydrogels for irradiation and duration time dual‐controlled dynamic decryption process to read the correct code.

In addition to being an effective photo‐rewritable material, the more fascinating feature is its kinetics‐controllable photochromic property. As confirmed above, CB[8] can act as a supramolecular catalyst for the photodimerization of CB[8]⊃**M**_2_. Here we further studied the kinetic effect of different amount of CB[8] on the photoreaction. As shown in **Figure** **6**A, three typical dynamic curves were measured for the mixture solutions with different component ratios, in which the absorption intensities at 390 nm against the irradiation time for **M** solutions exhibited different decay rates. In other words, distinct absorbance could be achieved at a given irradiation time for those “supramolecular cocktails” containing varied host–guest ratios between CB[8] and **M**. Therefore, if the absorbance greater than a threshold (for example, *A* = 0.5 in Figure 6A) is defined as the output signal “1,” while that less than the threshold is defined as an output signal of “0,” then for the “supramolecular cocktails” developed here, signals “0” and “1” may involve a time‐factor, i.e., different signals could be read out for a single cocktail at different time, on the other hand, different signals could also be observed for diverse cocktails at a certain time. Through this time‐involved dimension‐increase strategy, a normal digital sequence could be extended to a matrix‐like data with richer information (Figure 6B).

The dynamically adjustable photochromic properties of the CB[8]⊃**M**_2_ system further endow their intriguing potential in time‐resolved information encryption, which is superior to the traditional static technology. In essence, traditional encryption materials only require the appropriate physical technology or equipment to read the encrypted information, by which a definite result could be obtained. Whereas, using an encrypted material that contains the time‐factor will greatly increase the complexity of decryption, which not only requires the proper decryption technology, but also calls for the correct operating time, that is, one cannot get the right information by either longer or shorter decryption time. For example, we have demonstrated that the CB[8]⊃**M**_2_ system can undergo photodimerization in the hydrogel state, leading to a yellow‐to‐colorless transition, whose fading time could be finely tuned by different host–guest ratios. Based on this mechanism, as schematically illustrated in Figure 6C, a series of hydrogels containing different “supramolecular cocktails” (same concentrations of **M** while with different concentrations of CB[8]) could be constructed with similar yellow color in their initial states. By using these hydrogels as small sticks, an encrypted Arabic numeral code could be shaped on a plane. Due to the different kinetic characteristics of the photochromic hydrogels, the small sticks in this number can be decolorized at distinct time when irradiated by 365 nm UV light, thus presenting time‐resolved dynamic codes. As a preliminary practical application of this strategy (shown in Figure 5D), a yellow four‐digit number is made confidentially by using hydrogels containing different “supramolecular cocktails”. When irradiated by UV light, a series of different codes could be read out by the naked eye at the duration time of 0, 20, and 180 s, respectively, in which the correct information could only be obtained at the right time node (20 s), as marked by the green tick. As a result, this supramolecular photochromic system presents a convenient, low‐cost and time‐resolved dynamic encryption/decryption strategy, and the different information can be distinguished and read by the naked eye, providing a portable and close‐to‐life anticounterfeiting technology.

## 3 Conclusion

In summary, we have successfully developed a new ternary supramolecular complex CB[8]⊃**M**_2_ in aqueous solution by taking advantage of the synergetic noncovalent interactions, including cation‐*π* interactions, charge–dipole affinities, hydrophobic effects, *etc*. A kind of negative photochromism was realized for the supramolecular complex due to the reversible photodimerization/cleavage reactions of the chromophore **M** inside the cavity of CB[8]. Thanks to the preorganization and spatial confinement effects of the self‐assembly system, this photochromic material exhibits several excellent properties, such as, i) extremely rapid response time (*t*_1/2_ = 0.97 and 15.05 s for the forward and reverse reactions, respectively), ii) high conversion rate (98% and 95% for both the reactions respectively), and iii) high stereoselectivity of the photodimerized product. What's more, the macrocycle CB[8], acts as a supramolecular catalyst, can finely control the kinetics of the photodimerization of the host–guest system in aqueous or quasi‐solid phase, providing the material with tunable time‐dependent dynamic features, which could not only be applied to extend the normal digital sequence to a matrix‐like data with richer information, but also fabricate confidential materials with time‐resolved multidimensional dynamic encryption. This strategy is like opening a lock that requires two necessary keys, in which the “time fact” acts as a dynamic key that has multiple and unpredictable possibilities, making it more difficult to crack. We believe the present supramolecular system demonstrates a concise yet powerful platform and provides a guiding strategy for the construction of kinetically controllable photochromic materials, which could serve as ideal candidates for the potential application in time‐resolved information display and multidimensional dynamic encryption.

## Conflict of Interest

The authors declare no conflict of interest.

## Supporting information

Supporting Information

Supporting Information

Supporting Information

Supplemental Movie 1

## Data Availability

The data that support the findings of this study are available in the supplementary material of this article.
