# Supplementary material for: Time‐Resolved Encryption via a Kinetics‐Tunable Supramolecular Photochromic System
Source: Adv Sci (Weinh). 2022 Jan 6;9(6):2104790. doi: 10.1002/advs.202104790 (PMC8867189; doi:10.1002/advs.202104790)
Supplement: Supplementary file 1 — Supporting Information [file ADVS-9-2104790-s002.pdf]

ADVANCED SCIENCE
Open Access

Supporting Information

for *Adv. Sci.*, DOI: 10.1002/advs.202104790

Time-Resolved Encryption via a Kinetics-Tunable Supramolecular Photochromic System

Dong Li, Zefen Feng, Yujie Han, Chen Chen, Qi-Wei Zhang, and Yang Tian**

Supporting Information

Time-Resolved Encryption via a Kinetics-Tunable Supramolecular Photochromic System

Dong Li, Zefen Feng, Yujie Han, Chen Chen, Qi-Wei Zhang,* and Yang Tian*

Shanghai Key Laboratory of Green Chemistry and Chemical Processes, Department of Chemistry, School of Chemistry and Molecular Engineering, East China Normal University, Shanghai 200241, P.R. China.

Contents

1. Materials, general procedures, and synthesis. ---S2

2. Characterizations and studies on supramolecular systems. ----------------------------S5

3. References. ---S12

1. Materials, general procedures and synthesis.

1.1 Materials. Unless stated specifically, all reagents were purchased from Energy Chemical Co. Ltd (Shanghai) or 9DingChem Co. Ltd (Shanghai) and used without further purification. Solvents were purified by standard methods.

1.2 General. ^{1}H NMR, ^{13}C NMR, and 2D ROESY NMR spectra were measured on a Bruker AVIII-600 spectrometer. The electronic spray ionization (ESI) high resolution mass spectra (HRMS) were obtained on a Thermo Scientific Q Exactive mass spectrometer. The optical absorption measurements were carried out using a Hitachi UH-5300 absorption spectrophotometer. The emission spectra were measured on a Hitachi F-4600 fluorescence spectrometer. Isothermal titration calorimetry (ITC) experiment was carried out on a Ta at 25 °C. Both of the power of portable 365 nm UV light and 254 nm UV light were 5 W.

1.3 Synthesis

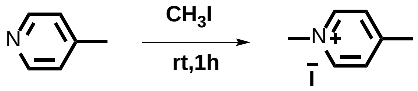

1

Synthesis of 1,4-dimethylpyridin-1-ium iodide (1).[S1] Iodomethane (2 mL, 32 mmol) was added to the solution of picoline (4 mL, 20 mmol) dissolved in ethylacetate (5 mL). The mixture was stirred at room temperature for 1 h. The precipitation was collected by filtration, washed with ethylacetate to obtain the product **1** as a yellow solid (4.28 g, 90% yield). ^{1}H NMR (600 MHz, D_2O) δ 8.47 (d, *J* = 5.7 Hz, 2H), 7.75 (d, *J* = 4.7 Hz, 2H), 4.21 (s, 3H), 2.55 (s, 3H)

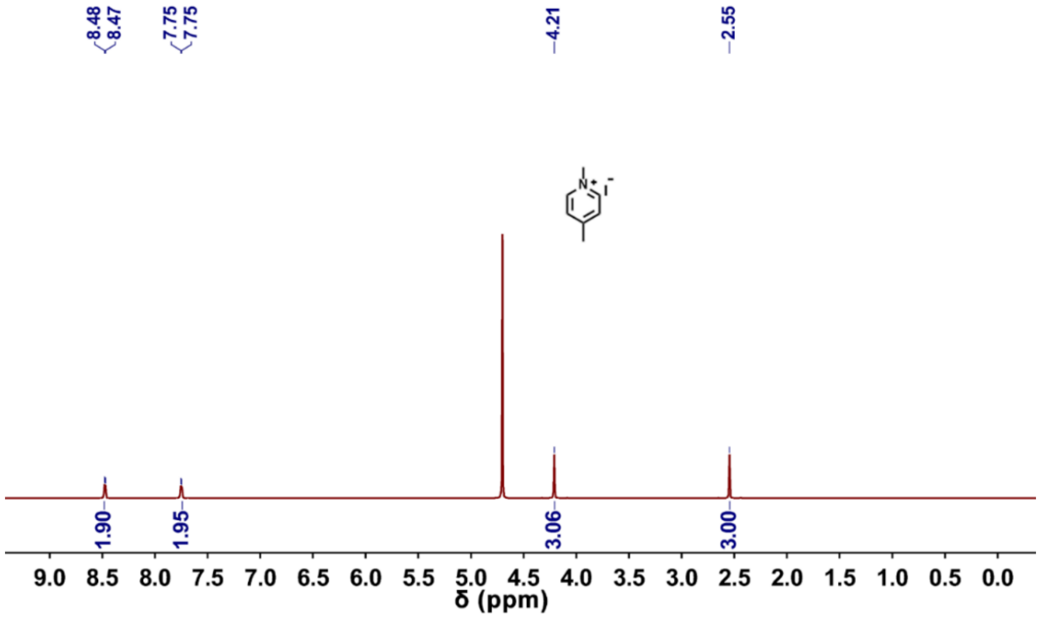

^{1}H NMR Spectrum of Compound **1** in D_2O, at 298K.

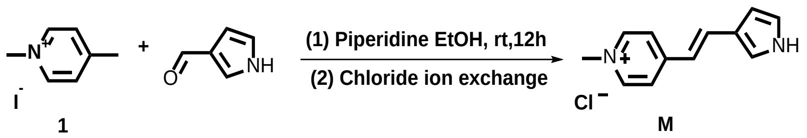

Synthesis of (E)-4-(2-(1H-pyrrol-3-yl)vinyl)-1-methylpyridin-1-ium chloride (M).[S2] Compound **1** (0.47 g, 2 mmol) and Pyrrole-3-carbaldehyde (0.28 g, 3 mmol, 1.5 equiv.) were dissolved in 5 mL EtOH. A drop of piperidine was add to the mixture, then the mixture solution was stirred for 12 hours at room temperature. The precipitation was collected by filtration, washed with ethyl alcohol and tetrahydrofuran in sequence. The counteranion of the compound was exchanged to chloride to get product **2** as a yellow solid (0.33 g, 75% yield). ^{1}H NMR (600 MHz, D_2O) δ 8.25 (d, J = 6.5 Hz, 2H), 7.71 (d, J = 6.6 Hz, 2H), 7.65 (d, J = 16.0 Hz, 1H), 7.17 (s, 1H), 6.88 (s, 1H), 6.77 (d, J = 16.0 Hz, 1H), 6.51 (s, 1H), 4.03 (s, 3H). ^{13}C NMR (151MHz, D_2O) δ 154.46, 143.55, 135.96, 123.44, 122.31, 121.33, 121.03, 117.83, 105.38, 46.32. HRMS *m / z* calcd for $[C_{12}H_{13}N_2]^+$ ([**M** - Cl]$^+$) 185.1073, found 185.1077.

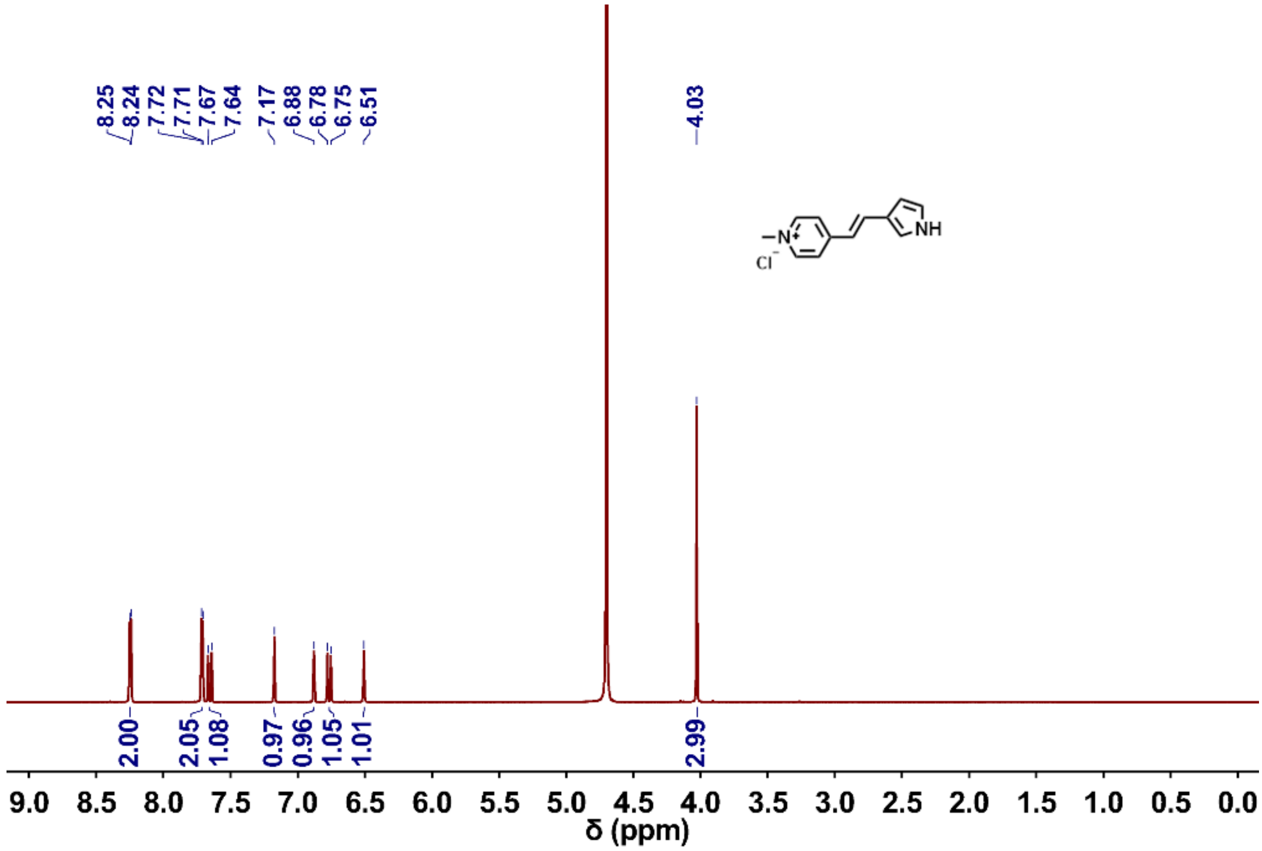

^{1}H NMR Spectrum of Compound **M** in D_2O, at 298K.

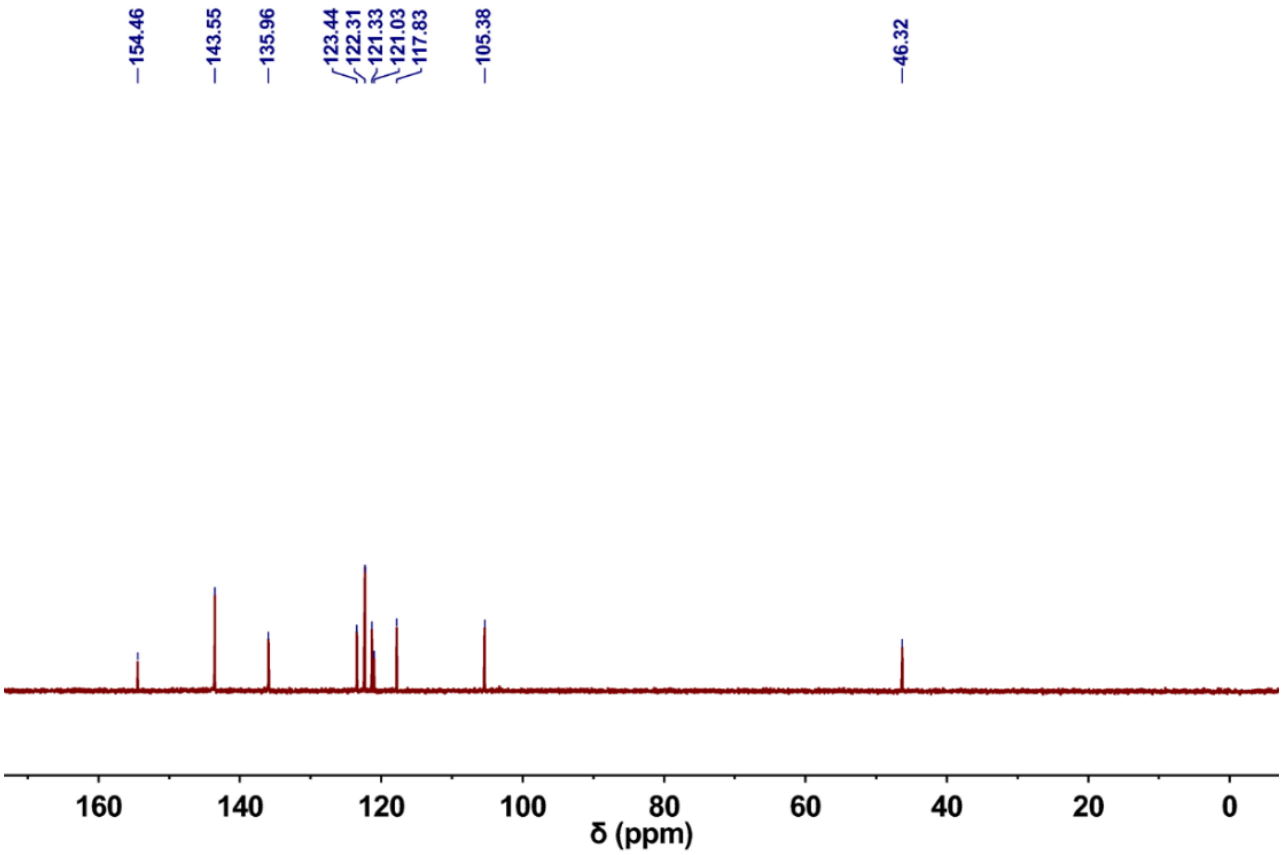

^{13}C NMR Spectrum of Compound **M** in D_2O, at 298K.

Acquisition Parameter

Source Type	ESI	Ion Polarity	Positive	Set Nebulizer	1.5 Bar
Focus	Active	Set Capillary	4500 V	Set Dry Heater	180 °C
Scan Begin	50 m/z	Set End Plate Offset	-500 V	Set Dry Gas	6.0 l/min
Scan End	1200 m/z	Set Collision Cell RF	800.0 Vpp	Set Divert Valve	Waste

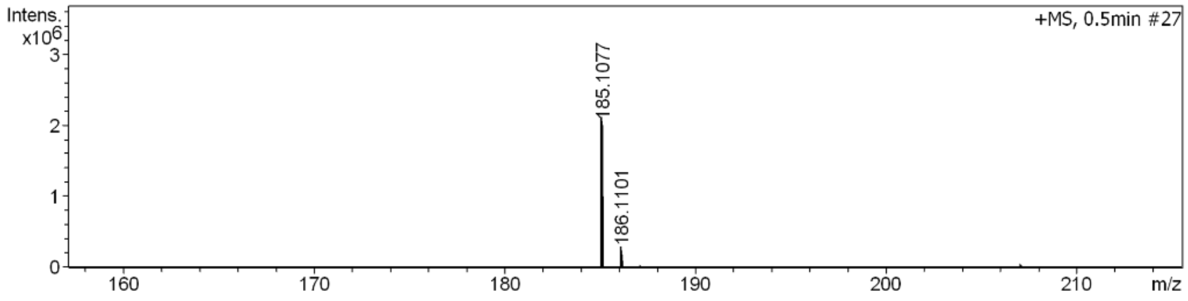

#	m/z	Res.	S/N	I	I %	FWHM
1	185.1077	9381	4280.7	2002036	100.0	0.0197
2	186.1101	10813	489.1	228872	11.4	0.0172
3	187.1132	11740	36.9	17308	0.9	0.0159

Meas. m/z	#	Ion Formula	m/z	err [ppm]	mSigma	#	Score	rdb	e⁻ Conf	N-Rule
185.1077	1	C12H13N2	185.1073	-2.1	14.0	1	100.00	7.5	even	ok

Mass Spectrum of Compound **M**

2. Characterizations and studies on the supramolecular systems.

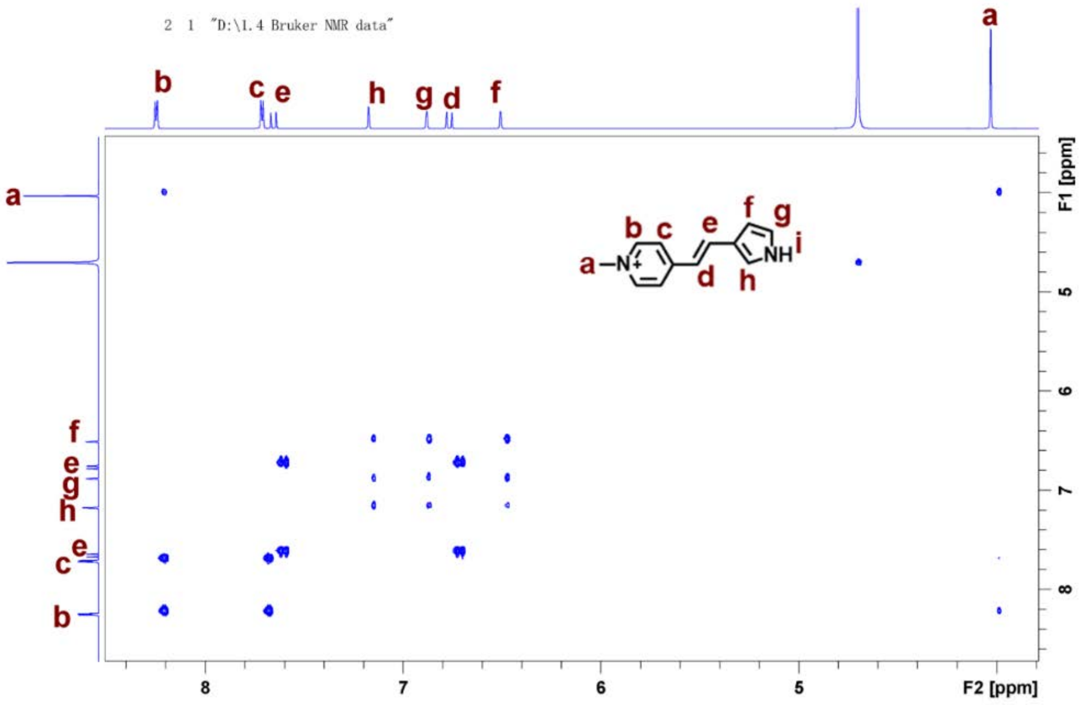

Figure S1. 2D COSY NMR spectrum of **M** (2 mM) in D_2O at 298K.

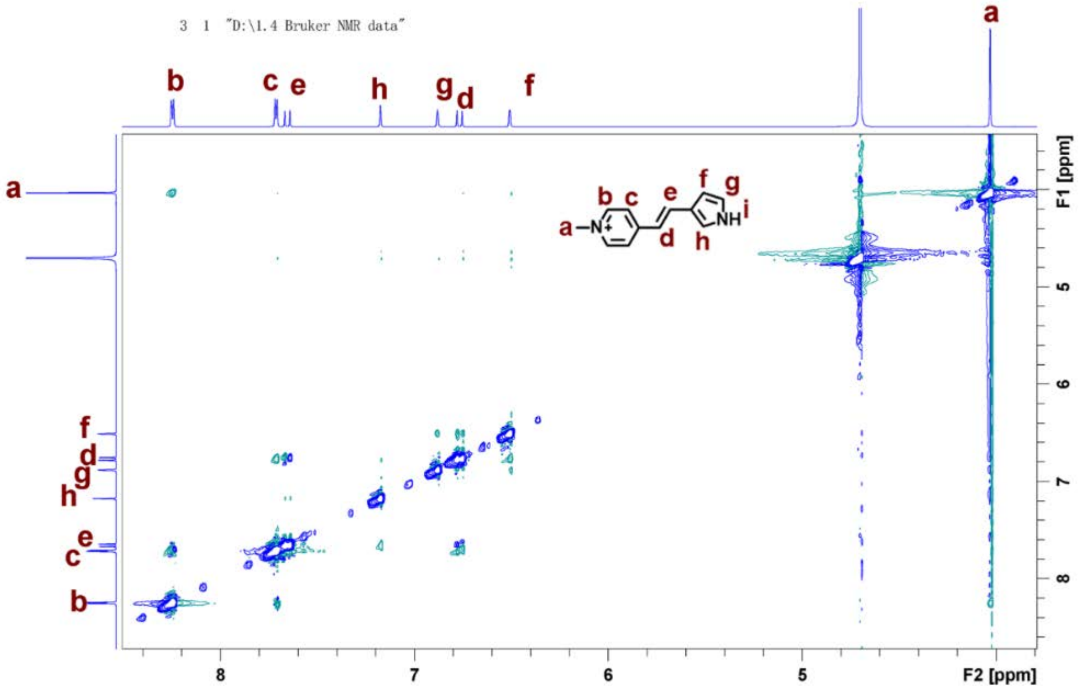

Figure S2. 2D ROESY NMR spectrum of **M** (2 mM) in D_2O at 25 298K.

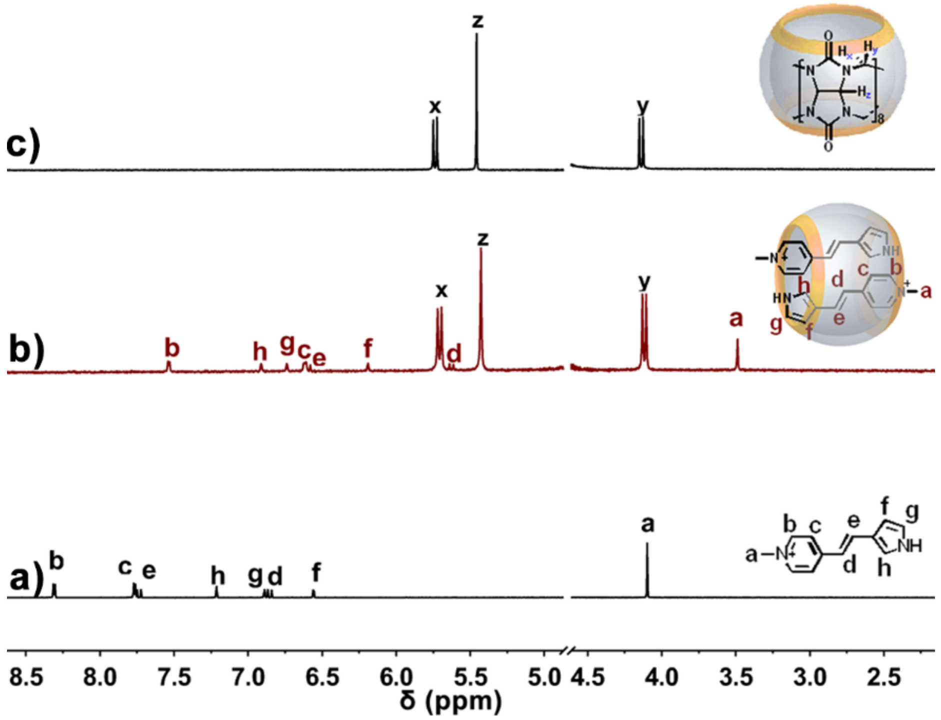

Figure S3. ^{1}H NMR spectra of **M** ([**M**] = 2 mM) (a), **M**+0.5 equivalent of CB[8] (b), and CB[8] (c) in D_2O at 25 °C.

Table S1. Crystal data and structure refinement for **M** (CCDC 2103177).

Empirical formula	$C_{24}H_{28}I_2N_4O$
Formula weight	642.30
Temperature/K	100.00(10)
Crystal system	monoclinic
Space group	$P2_1/n$
a/Å	10.28510(10)
b/Å	19.5784(2)
c/Å	13.06390(10)
α/°	90
β/°	101.6790(10)
γ/°	90
Volume/Å^3	2576.16(4)
Z	4
ρ_{calc}g/cm^3	1.656
μ/mm^{-1}	19.342

F(000)	1256.0
Crystal size/mm^3	0.32 × 0.28 × 0.12
Radiation	CuKα (λ = 1.54184)
2Θ range for data collection/°	9.876 to 134.144
Index ranges	-10 ≤ h ≤ 12, -23 ≤ k ≤ 23, -15 ≤ l ≤ 14
Reflections collected	25481
Independent reflections	4589 [R_{int} = 0.0519, R_{sigma} = 0.0361]
Data/restraints/parameters	4589/0/290
Goodness-of-fit on F^2	1.021
Final R indexes [I>=2σ (I)]	R1 = 0.0261, wR2 = 0.0611
Final R indexes [all data]	R1 = 0.0298, wR2 = 0.0625
Largest diff. peak/hole / e Å^{-3}	0.96/-0.66

Table S2. Crystal data and structure refinement for CB[8]⊃**M**$_2$ (CCDC 2116478).

Empirical formula	$C_{72}H_{82}Cl_2N_{36}O_{20}$
Formula weight	1842.63
Temperature/K	173.00(10)
Crystal system	monoclinic
Space group	$P2_1/n$
a/Å	26.4681(6)
b/Å	25.7767(4)
c/Å	13.0059(10)
α/°	90
β/°	90.437(2)
γ/°	90
Volume/Å^3	8873.2(3)
Z	4
ρ_{calc}g/cm^3	1.379
μ/mm^{-1}	1.412
F(000)	3840.0
Crystal size/mm^3	0.36 × 0.32 × 0.28
Radiation	CuKα (λ = 1.54184)

2Θ range for data collection/°	6.858 to 134.152
Index ranges	-31 ≤ h ≤ 25, -30 ≤ k ≤ 30, -15 ≤ l ≤ 15
Reflections collected	111890
Independent reflections	15789 [R_{int} = 0.0545, R_{sigma} = 0.0354]
Data/restraints/parameters	15789/40/1185
Goodness-of-fit on F^2	1.056
Final R indexes [I>=2σ (I)]	R1 = 0.1114, wR2 = 0.2910
Final R indexes [all data]	R1 = 0.1164, wR2 = 0.2945
Largest diff. peak/hole / e $Å^{-3}$	1.76/-1.24

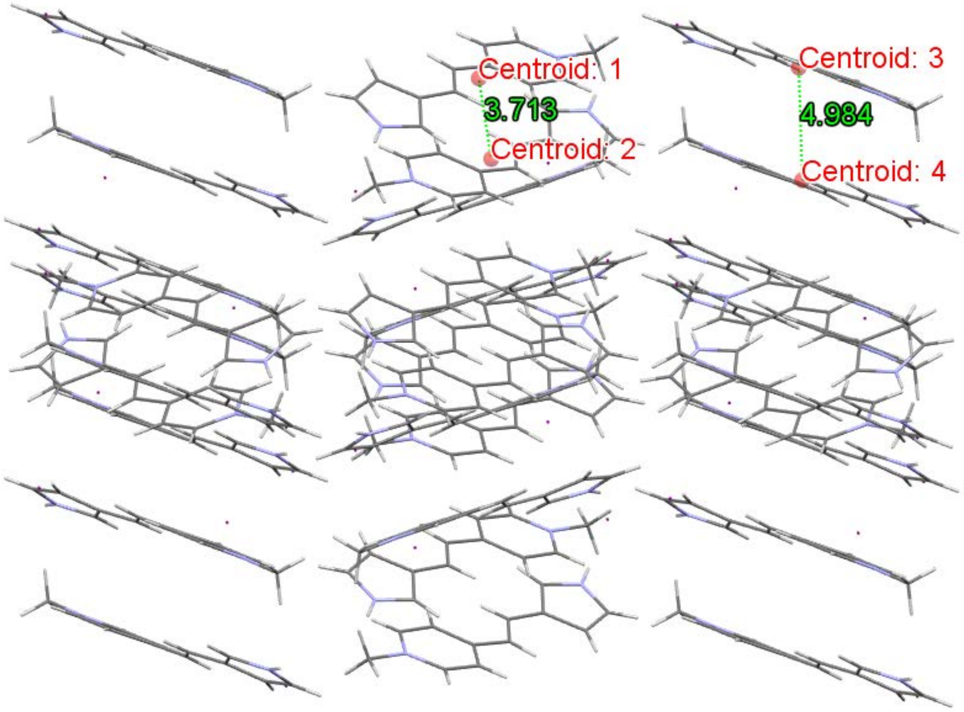

Figure S4. Molecular stacking view of the single crystal structure of **M**.

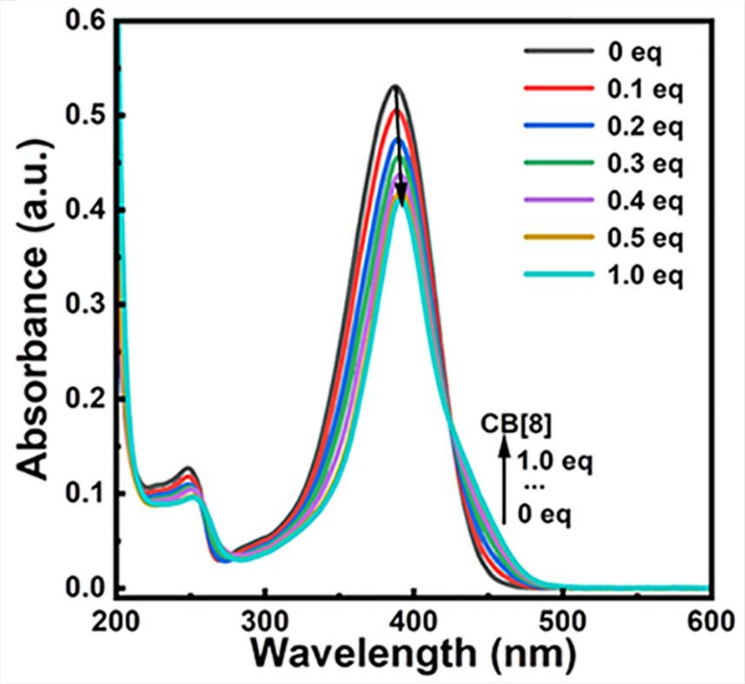

Figure S5. UV-Vis absorption spectra of **M** upon addition of different equivalences of CB[8] in aqueous solution, [**M**] = 20 μM.

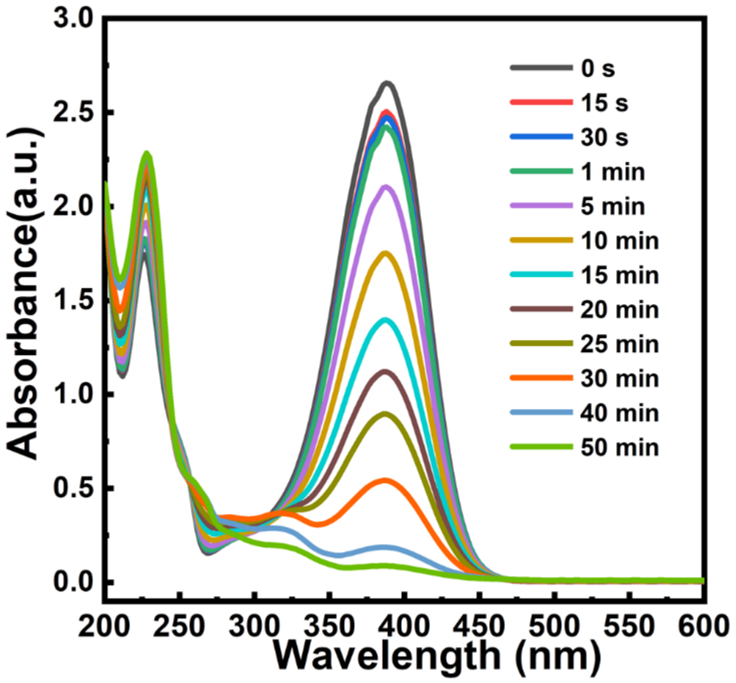

Figure S6. Absorption spectra changes of the aqueous **M** solution ([**M**] = 100 μM) upon irradiation with 365 nm UV light for different time (0 ~ 50 min).

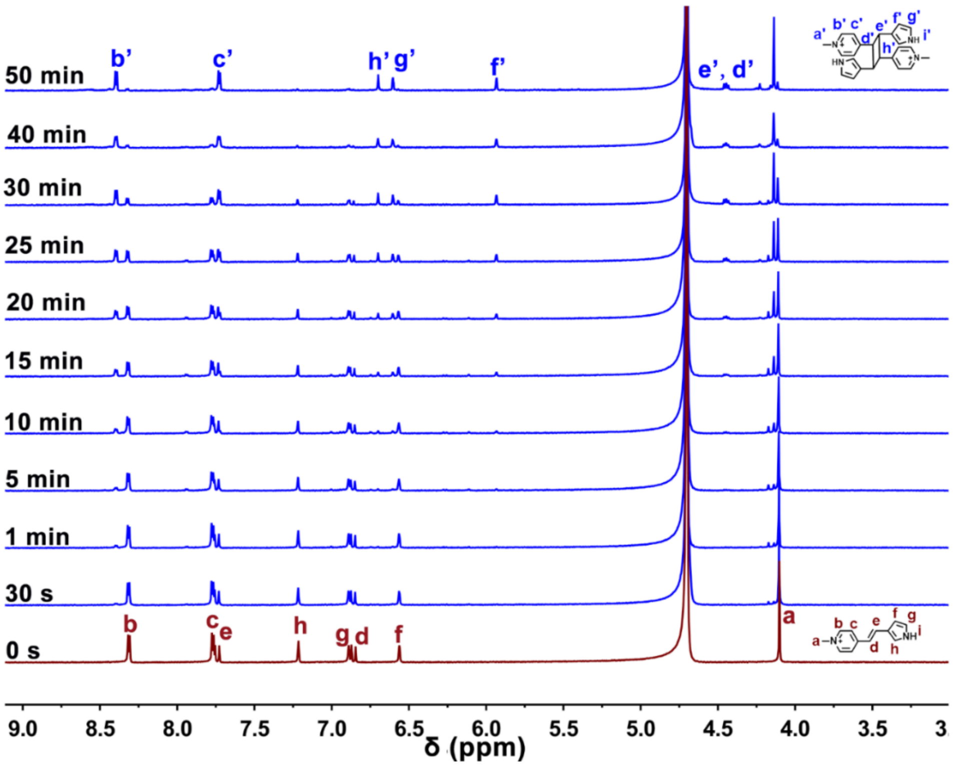

Figure S7. ^{1}H NMR spectra changes of **M** ([**M**] = 2 mM, in D_2O, at 298 K) upon irradiation with 365 nm UV light for different time (0 ~ 50 min).

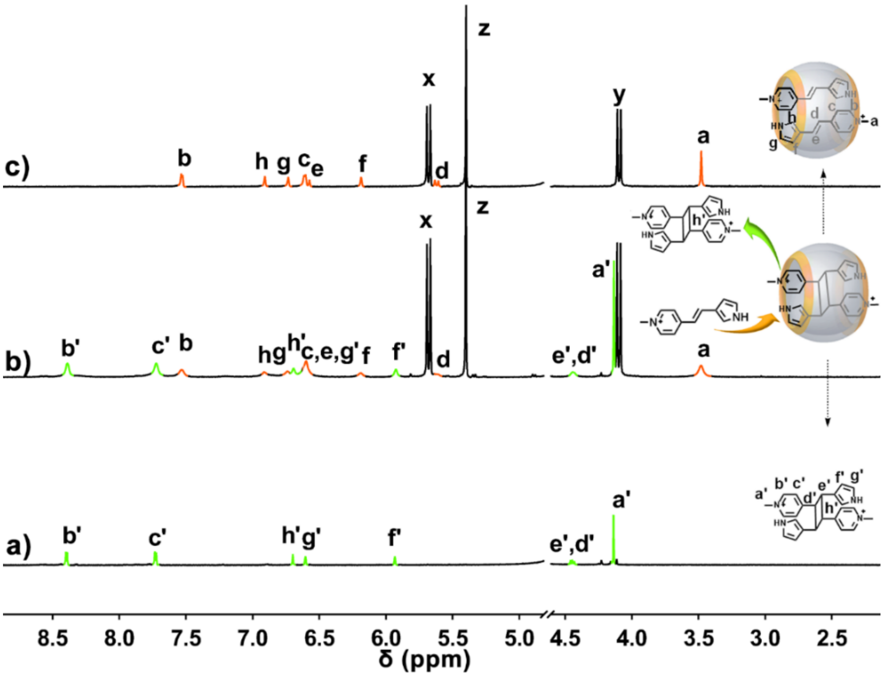

Figure S8. ^{1}H NMR spectra of the aqueous **D** solution ([**D**] = 1 mM) (a), the complex CB[8]⊃**D** ([**D**] = 1 mM) upon addition of a small amount of **M** (b), and the complex CB[8]⊃$\mathbf{M}_2$ ([**M**] = 2 mM) (c), in D_2O at 298 K.

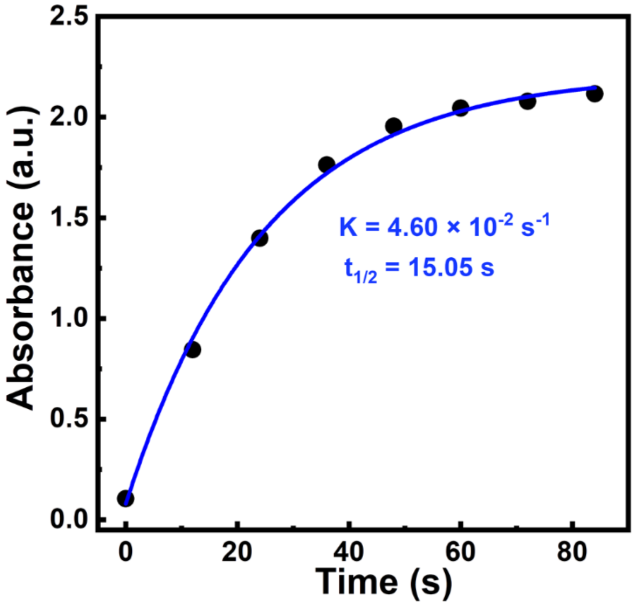

Figure S9. The absorbance intensity change of the aqueous CB[8]⊃**D** solution ([**D**] = 50 μM) at 390 nm (black circles) upon 254 nm UV light irradiation for different time, and fitted with the first-order kinetic model (blue line).

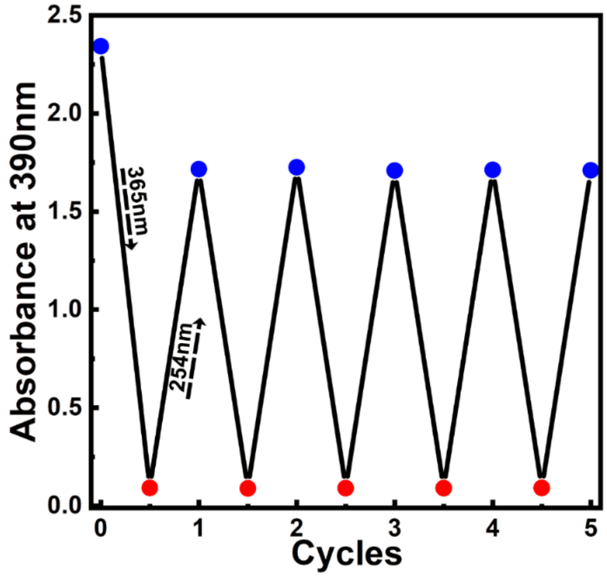

Figure S10. Intensity changes of the absorption at 390 nm for the complex CB[8]⊃$\mathbf{M}_2$ in aqueous solution upon alternating UV lights irradiations (365 nm, 10 s; and 254 nm, 35 s at 60 °C).

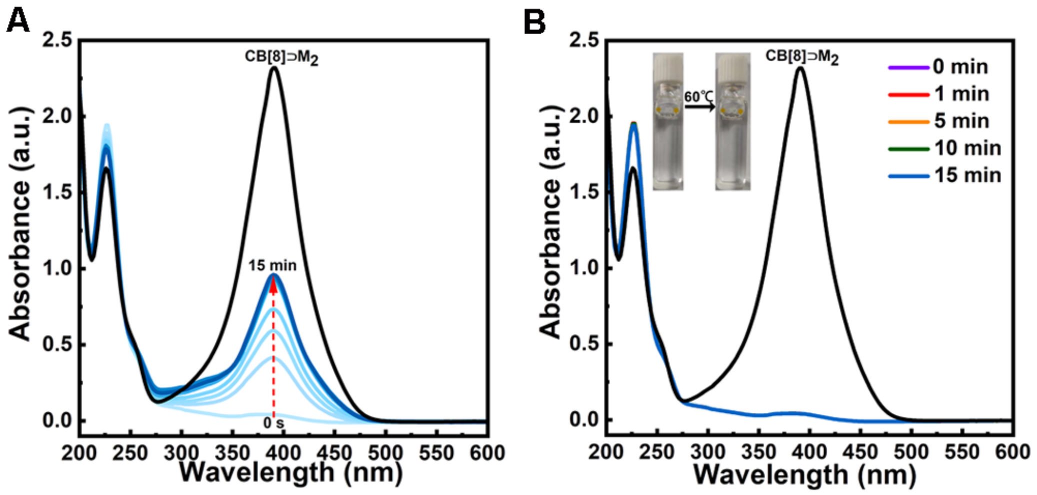

Figure S11. Absorption spectra changes of the aqueous CB[8]⊃**D** solution ([**D**] = 50 μM) upon irradiation with 254 nm UV light at room temperature (A), or heated but without 254 nm light irradiation (B), for 0-15 min.

3. References.

[S1] Guo, L.; Li, C.; Shang, H.; Zhang, R.; Li, X.; Lu, Yu, X.; *Chem. Sci.* **2020**, *11*, 661-670.

[S2] Jana, P.; Radhakrishna, M.; Khatua, S.; Kanvah, S.; Phys. *Chem. Chem. Phys.*, **2018**, *20*, 13263-13270.
